# Characteristics of ocular findings of patients with coronavirus disease 2019 in China

**DOI:** 10.3389/fmed.2024.1292821

**Published:** 2025-01-13

**Authors:** Shudan Wang, Jiaoyang Feng, Rui Zhu, Xin Jin, Yiheng Ding, Di Jin, Yu Guo, Hong Zhang

**Affiliations:** Eye Hospital, The First Affiliated Hospital of Harbin Medical University, Harbin, China

**Keywords:** COVID-19, ocular symptoms, survey study, systemic symptoms, retinal manifestations

## Abstract

**Purpose:**

This study aimed to report the ocular manifestations in individuals with coronavirus disease 2019 (COVID-19) and investigate any correlation between the occurrence of ocular symptoms and systemic symptoms.

**Methods:**

A retrospective electronic survey was conducted among the general public in northern China from December 2022 through February 2023. Inclusion criteria for COVID-19 was confirmed testing positive via a polymerase chain reaction (PCR) test or testing positive for COVID-19 via an antigen kit. The anonymous survey collected information on demographics, systemic COVID-19 symptoms, ocular diagnosis and symptoms, comorbidities and disease history.

**Results:**

A total of 2,405 survey responses were collected and the final analysis included individuals in the 335 COVID-19 positive group and 434 individuals in the COVID-19 negative group. Among COVID-19 positive patients 66.3% (*n* = 222) reported experiencing ocular symptoms. Dryness (*n* = 106, 31.6%), blurred vision (*n* = 81, 24.2%), eye pain (*n* = 72, 21.5%), and itching (*n* = 71, 21.2%) were the main features. COVID-19 was found to be associated with a higher prevalence of conjunctivitis, iritis, uveitis, retinal vein occlusion and optic neuritis. The majority of individuals (51.0%) developed eye symptoms after the onset of COVID-19 systemic symptoms. There was no significant association between the severity of systemic symptoms and ocular symptoms.

**Conclusion:**

Individuals with COVID-19 were significantly more likely to experience ocular symptoms. COVID-19 was found to be associated with a higher prevalence of retinal diseases. The majority of individuals developed ocular symptoms right after the onset of systemic symptoms.

## Introduction

Severe acute respiratory syndrome coronavirus 2 (SARS-CoV-2) is a novel enveloped, positive single-stranded RNA beta coronavirus that causes coronavirus disease 2019 (COVID-19) (1). While respiratory symptoms and myalgias are the primary clinical features, recent reports suggest the involvement of the eyes as well (2–5). Evidence shows that the virus can be detected in tears, suggesting that the ocular surface may serve as a potential entry point and reservoir for transmission (2). Clinically, a number of ocular manifestations have been reported to be associated with SARS-CoV-2, including conjunctival hyperemia or chemosis, epiphora, ocular pain, itching, blurred vision and increased secretions (3, 5). The prevalence of ocular manifestations in patients with COVID-19 ranges from 2 to 32%, with conjunctivitis considered the most common ocular manifestation (4). There have been a few studies investigating the impact of SARS-CoV-2 on ocular manifestations, which initially focused on conjunctivitis, and then gradually there were some case reports of retinal manifestations believed to be related to COVID-19 infection (6–9). Although the epidemic caused by COVID-19 is now behind us, reflecting on it and learning from the experience can help prevent a recurrence in the future. When the epidemic isolation policy was canceled, our doctors found that after being infected with COVID-19, in addition to the manifestations of conjunctivitis, there are many other eye diseases, including keratitis, iritis, scleritis, uveitis, retinal vein occlusion and optic neuritis. In addition, the majority of published reports describing ocular symptoms involve hospitalized patients, whereas comparatively less is known about those treated in the outpatient setting. To address this gap, our study aims to fill an important knowledge gap regarding the ocular manifestations of COVID-19 and contribute to a more comprehensive understanding of the disease’s impact on the eye. This would provide valuable information on the frequency, timing, extent, and duration of SARS-CoV-2-associated ocular symptoms in a broader population.

## Methods

This retrospective electronic survey was designed to gather a large amount of information regarding ocular diseases and symptoms during the COVID-19 pandemic in northeast China. The study information was distributed to the general public through social media by a questionnaire. The contents of the questionnaire include: basic information of the participants, whether they had been infected with SARS-CoV-2, details of their SARS-CoV-2 infection, COVID-19 vaccination situation, past ocular and medical history, ocular diseases and symptoms after infection with SARS-CoV-2. All responses were kept anonymous, and survey responses were collected from December 2022 through February 2023. In total of 2,405 individuals participated in the survey. Only complete responses were included for analysis. Those who had never done relevant tests to confirm COVID-19 were excluded. We counted 1,589 responders, accounting for 66.0%. To avoid other factors influencing the analysis, we also excluded respondents who had hypertension, diabetes and rheumatic system disease and patients with preexisting eye disease. We ended up with 769 participants. The criteria for judging the infection of SARS-CoV-2 as follows: confirmed testing positive via a polymerase chain reaction (PCR) test or testing positive for COVID-19 via an antigen kit. Those who tested negative were negative control. The study was approved by the Ethics Committee of the First Affiliated Hospital of Harbin Medical University, and registered with China Clinical trial Center (IRB-AF/SC-05/04.0). All procedures followed the tenets of the Declaration of Helsinki.

For statistical analyses, SPSS v.17.0 statistical software for Windows (SPSS Inc., Chicago, IL) was used. For descriptive statistics, continuous variables are presented as means ± standard deviations, and categorical variables are presented as frequencies and proportions. Multivariable logistic regression models were used to identify significant predictors of clinical symptoms adjusted by age, gender and race. Comparisons of continuous variables between two variables were done by student t-test for normally distributed variables. For variables without normal distribution, Mann–Whitney U test was used for comparisons between two independent variables. Two-way ANOVA was used to compare the effects of two categorical variables on an outcome variable. Correlation analysis was used to measure the relationship between two continuous variables, using correlation coefficients such as Pearson’s correlation coefficient. A two-tailed *p* value of < 0.05 was considered significant.

## Results

Based on the inclusion and exclusion criteria outlined in methods, we obtained a final population of 335 individuals in the COVID-19 positive group and 434 individuals in the COVID-19 negative group. The demographic characteristics are summarized in Table 1.

**Table 1:** Participant demographics.

		Tested positive	Tested negative
Total, *n*		335	434
Sex, *n* (%)			
	Female	221 (65.8)	245 (57.7)
	Male	114 (34.2)	189 (42.3)
Age, years			
	Range	1–79	1–87
	Mean ± SD	39.6 ± 14.4	40.6 ± 17.6
Province, *n* (%)			
	Heilongjiang	306 (91.3)	418 (96.3)
	Others	29 (8.7)	16 (3.7)
With COVID-19 Symptoms, *n* (%)		299 (89.3)	70 (16.1)
With ocular symptoms, *n* (%)		222 (66.3)	256 (59.0)

COVID-19 symptoms included fever, loss of smell, unusual muscle pains, headache or dizzy, lethargy, abdominal pain or diarrhea and others.

Ocular symptoms included dryness, itchy, ocular pain, redness, photophobia and tears, blurred vision, swelling, increased discharge and others.

**Figure 1:**
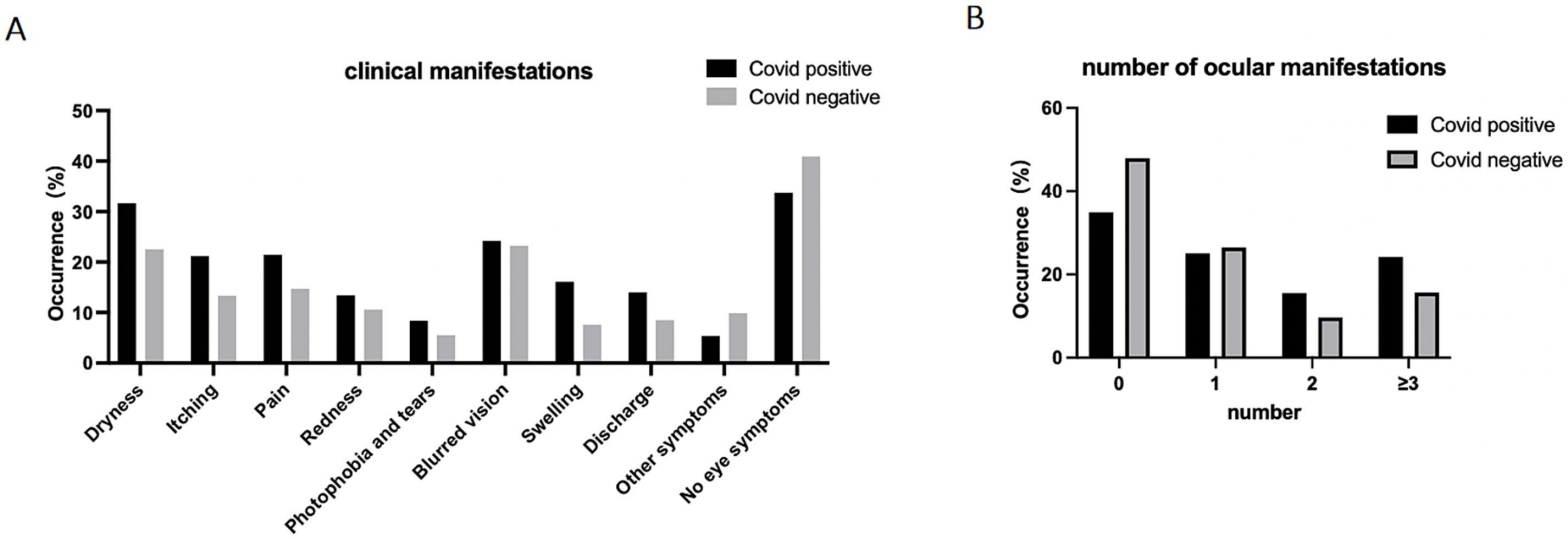
Ocular manifestation of participants. Data are presented as percentage of participants who reported experiencing ocular symptoms. **(A)** Prevalence of ocular symptoms reported by individuals, data are presented as percentage of participants. **(B)** Number of reported eye symptoms experienced by participants, data are presented as percentage of participants.

Among the individuals who tested positive for COVID-19, a total of 222 people (66.3%) reported experiencing ocular symptoms. In the COVID-19 negative group, 256 people (59.0%) reported ocular symptoms. The most commonly reported ocular symptoms among COVID-19 positive participants were dryness (*n* = 106, 31.6%), blurred vision (*n* = 81, 24.2%), pain (*n* = 72, 21.5%), and itching (*n* = 71, 21.2%), as depicted in Figure 1. The COVID-19 positive group had a higher percentage of each symptom compared to the negative group. Furthermore, the positive group had a higher proportion of individuals experiencing multiple symptoms. Specifically, 24.2% (*n* = 81) of positive participants reported having three symptoms or more than that, while this was the case for 15.7% (*n* = 68) of negative participants.

Then, we conducted an analysis to examine the relationship between specific ocular symptoms and COVID-19, and the results are summarized in Table 2. The table shows significant associations between COVID-19 and some ocular symptoms. The presences of dryness, itchy, ocular pain, eye swelling, and increased eye discharge showed statistically significant positive association with COVID-19, with odds ratios of 1.587, 1.743, 1.583, 2.335, and 1.751, respectively. On the other hand, symptoms like red eyes and photophobia and tearing do not show a statistically significant association with COVID-19, as their *p*-values are higher than the predefined significance level. The table provides valuable information on the potential ocular symptoms that may be linked to COVID-19 and can aid in understanding the ocular manifestations associated with the disease. We also analyzed the association between specific systemic symptoms and COVID-19 which is summarized in Supplementary Table 1. Individuals with COVID-19 were significantly more likely to experience symptoms such as fever, decreased sense of taste or smell, muscle pain, headache, drowsiness, and abdominal pain.

**Table 2:** Relationship between COVID-19 and ocular symptoms.

Ocular symptom	Symptom present	COVID-19 infection	OR	95%CI	*p*-value
Dryness	No	229 (40.5%)	1		
	Yes	106 (52.0%)	1.587	1.150–2.190	0.005
Itchy	No	264 (41.3%)	1		
	Yes	71 (55%)	1.743	1.191–2.552	0.004
Ocular pain	No	263 (41.5%)	1		
	Yes	72 (52.9%)	1.583	1.091–2.296	0.016
Redness	No	290 (42.8%)	1		
	Yes	45 (49.5%)	1.309	0.844–2.029	0.229
Photophobia and tears	No	307 (42.8%)	1		
	Yes	28 (53.8%)	1.558	0.886–2.741	0.124
Blurred vision	No	254 (43.3%)	1		
	Yes	81 (44.5%)	1.051	0.752–1.470	0.769
Eye swelling	No	281 (41.2%)	1		
	Yes	54 (62.1%)	2.335	1.475–3.696	<0.001
Increased eye discharge	No	288 (42%)	1		
	Yes	47 (56.0%)	1.751	1.109–2.765	0.016

**Figure 2:**
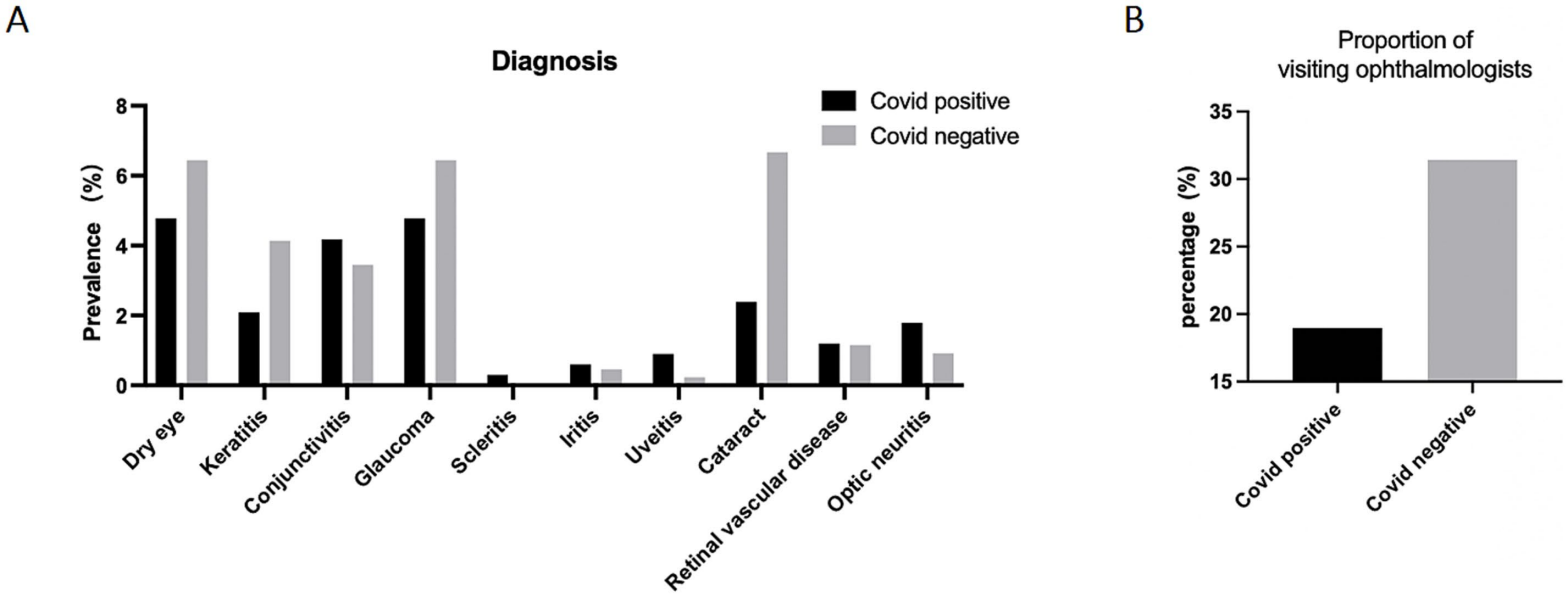
Ocular diagnosis of participants. **(A)** Ocular diagnosis of participants. Data are presented as percentage of participants who reported clinical ocular diagnosis. **(B)** The proportion of participants who visited ophthalmologists after developing ocular symptoms. Data are presented as percentage.

We analyzed ocular clinical diagnosis of patients who visited the hospital and were examined by an ophthalmologist. As shown in the Figure 2A, the COVID-19-positive group exhibited a significant higher percentage of the clinical diagnosis of conjunctivitis, scleritis, iritis, uveitis, retinal vein occlusion and optic neuritis (ON) compared to the negative group. It was worth noting that the proportion of patients who sought timely medical attention after the onset of ocular symptoms was nearly two-times in the COVID-19 negative group compared to the COVID-19 positive group (Figure 2B).

**Figure 3:**
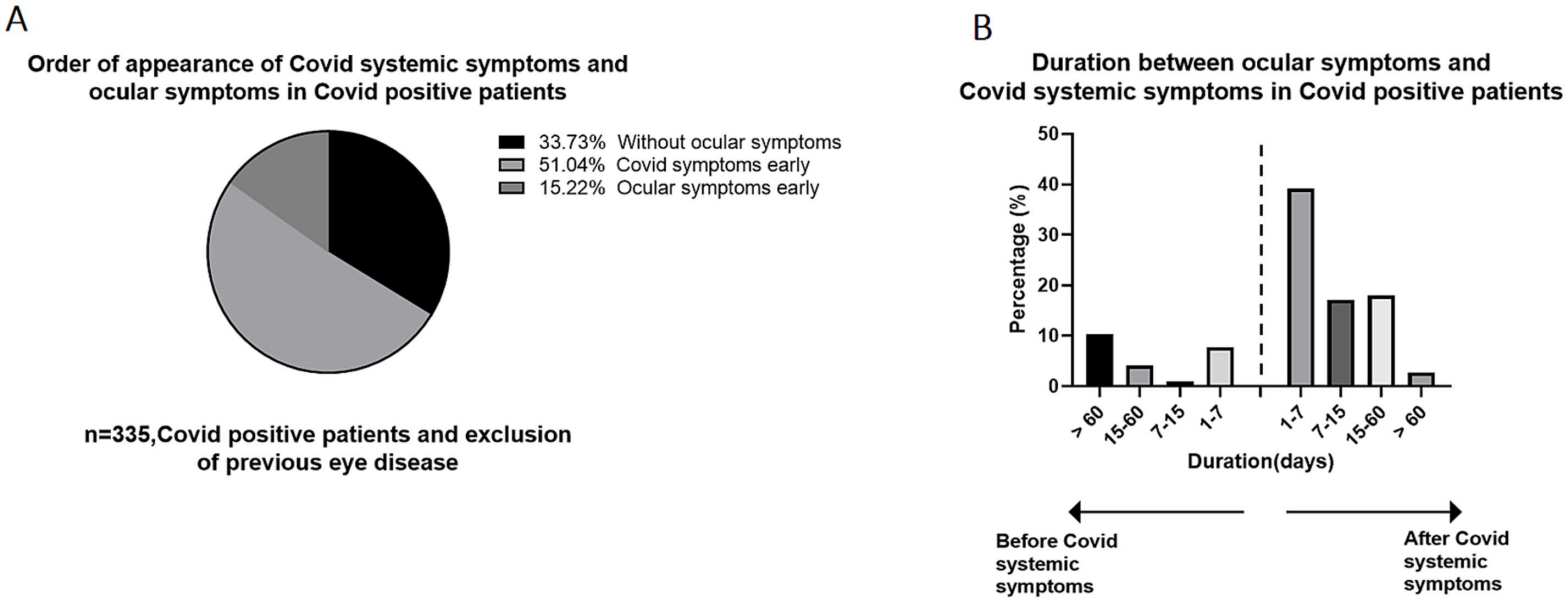
The time interval between ocular manifestation and systemic manifestation occurrence in COVID-19 positive patients. **(A)** The order of appearance of systemic symptoms and ocular symptoms in Covid positive patients. 51.0% of individuals developed ocular symptoms after the onset of COVID-19 systemic symptoms. **(B)** The time interval between ocular manifestation and systemic manifestation occurrence in COVID-19 positive patients, patients developed ocular symptoms 1–7 days after systemic symptoms accounted for the largest proportion, accounting for 39.2%.

**Figure 4:**
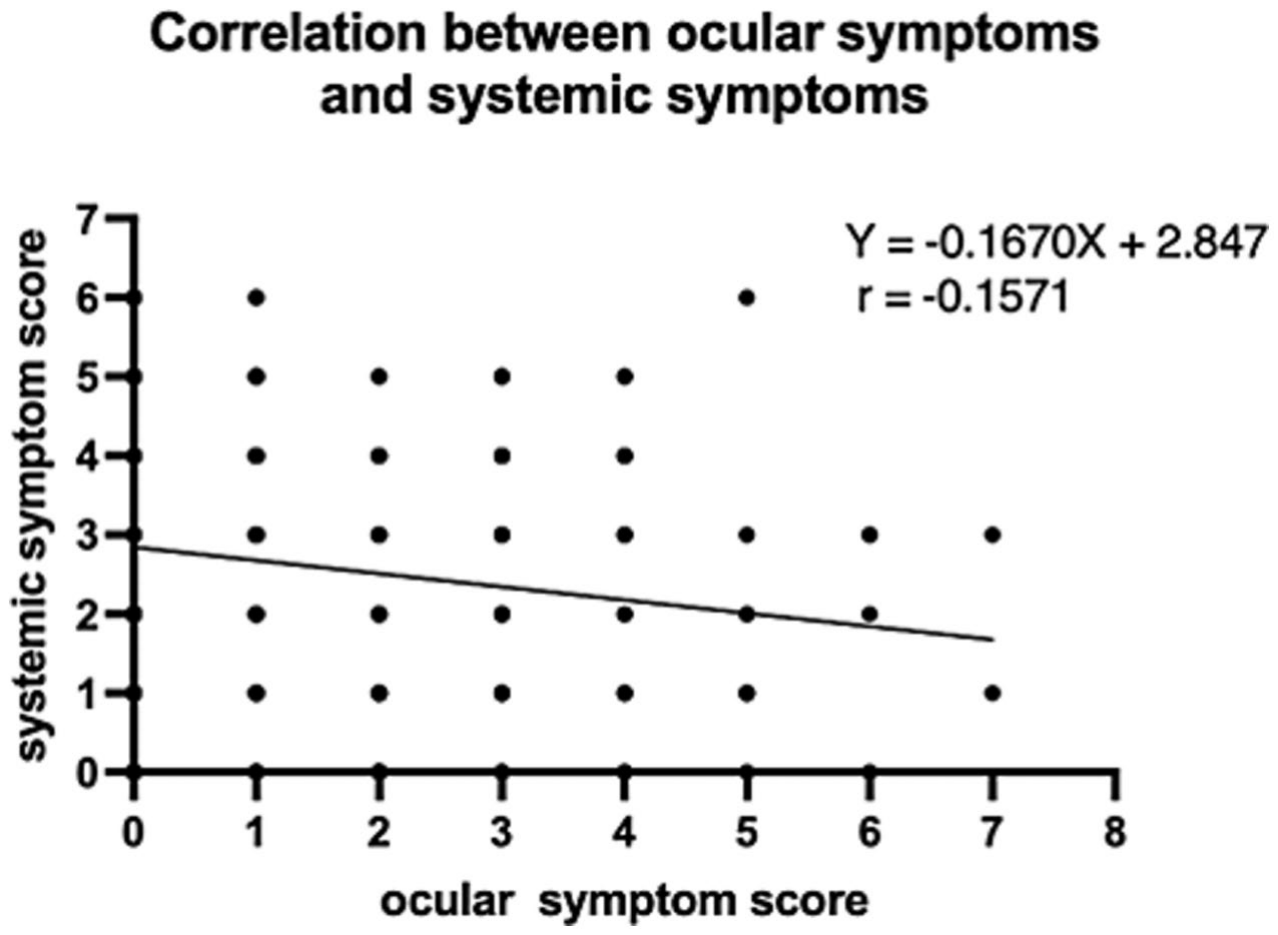
Correlation between ocular symptoms and systemic symptoms in COVID-19 positive patients. No Significant correlation between ocular symptom score and systemic symptom score.

We further delved deeper into the sequence in which ocular symptoms and systemic symptoms appeared. Among the COVID-19-positive participants, 171 participants (51.0%) developed systemic symptoms first, followed by ocular symptoms. Conversely, 51 participants (15.2%) reported having ocular symptoms before experiencing systemic symptoms (Figure 3A). We also investigated the time interval between the appearance of ocular symptoms and systemic symptoms, as depicted Figure 3B. The majority of individuals, comprising 87 participants (39.2%), developed eye symptoms 1–7 days after the onset of COVID-19 systemic symptoms. The second largest group experienced ocular symptoms between 15 and 60 days following the onset of systemic symptoms. There is also a high population also reporting experiencing ocular symptoms more than 60 days before the onset of systemic symptoms, which could potentially be attributed to preexisting eye conditions unrelated to the current illness. It is worth noting that regarding whether the anterior segment manifestations precede the posterior segment manifestations, we found that whether it is retinal vascular disease or optic neuritis, ocular symptoms appear after systemic symptoms, but in dry eye, keratitis and conjunctivitis, a higher proportion of individuals experience ocular symptoms before systemic symptoms. Moreover, we conducted an analysis to determine the correlation between the total score of systemic symptoms and ocular symptoms, and the results revealed that there is not a significant relationship between the two (Figure 4).

Finally, we also investigated the influence of vaccine on ocular symptoms, Among the 335 positive patients, the majority of patients received either 2 or 3 doses of the vaccine, the study primarily focused on analyzing the difference between these two groups. As shown in Supplementary Figure 2, the probability of ocular symptoms in positive patients who received 2 doses of the vaccine (*n* = 50) was reported to be 66.0% (33 patients). Similarly, the probability of ocular symptoms in positive patients who received 3 doses of the vaccine (*n* = 253) was reported to be 64.4% (163 patients).

## Discussion

Our findings reveal several noteworthy points, including (1) individuals with COVID-19 exhibited a higher incidence of ocular symptoms compared to the negative group; (2) COVID-19was found to be associated with a higher prevalence of retinal diseases; and (3) ocular symptoms are more likely to manifest shortly after the onset of systemic symptoms.

A recent meta-analysis by Soltani et al. found that the most prevalent ocular symptoms were dry eyes (23.8%) and eye pain (10.3%) (10). Another meta-analysis from Nasiri et al., they found the most common ocular manifestations to be dry eyes/foreign body sensation (16.0%), redness (13.3%), tearing (12.8%), and itching (12.6%) (11). Although varied, these analyses are largely consistent with our finding, the top ocular symptoms commonly complained by COVID-19 positive patients in our study, including blurred vision, dry eye, eye pain and itching.

In large-scale studies, the reported prevalence of ocular findings in COVID-19 positive patients have varied a lot (12, 13). The conjunctiva is considered to be one of the routes of infection of the new crown (14). Although SARS-CoV-2 RNA has recognition in tears and conjunctiva sac of patients with COVID-19, but the relationship between the transmission of SARS-CoV-2 and ocular symptoms remains unclear (15, 16). However, most of COVID-19 related eye diseases revolve around conjunctivitis, other eye diseases include uveitis, acute macular neuroretinopathy, and retinopathy (17, 18). Our results showed that the infection rate of conjunctivitis in COVID-19 patients was not as high as other studies, which may be related to the season, the patient information we collected is mainly concentrated in winter. In previous studies, the incidence of conjunctivitis was season-related and peaked in spring (19), which may explain our confusion above.

From the data we collected, compared to the control group, patients with COVID-19 showed higher infection rates of uveitis and optic neuritis (ON) compared with negative group. It was worth noting that patients with uveitis were immunocompromised, which increased their susceptibility to COVID-19 and experienced severe disease outcomes (20). Particularly those uveitis patients with ongoing biologic therapy and systemic corticosteroids, which was associated with greater risk of SARS-CoV-2 infection and severe outcomes in patients with noninfectious uveitis (20). In addition, COVID-19 pandemic led to reduce the follow-up compliance in uveitis patients, resulting in exacerbation of ocular symptoms (21). A few reports have been published till date describing ON during the recovery period from COVID-19 (22, 23). Few of them were associated with COVID-19-related vaccines associated with serum antibodies against myelin oligodendrocyte glycoprotein (MOG) (24, 25). More studies reported ON associated with COVID-19-related vaccines. In these cases, ON had unique symptoms and, in the majority of cases, resolved on its own without special care. Although some studies report COVID-19 vaccine may lead to eye diseases, including COVID-19 vaccine-associated uveitis, acute corneal graft rejection, post-vaccination retinal disease, optic neuritis and herpetic eye disease. However, these reports could not infer the causation and direct association from isolated cases (26–28). However, from the data we collected, ocular side effects following COVID-19 vaccination were very rare.

There are some case reports of COVID-19-associated glaucoma, but more research focuses on the management of glaucoma patients during the epidemic (29, 30). Özmen et al. reported three cases that developed glaucoma on the background of hyponatremia due to COVID-19 (31). Several studies also reported neovascular glaucoma and acute angle-closure glaucoma following COVID-19 (32, 33). It is intriguing to note that the prevalence of glaucoma is higher in COVID-19-negative group. This finding may be attributed, at least in part, to the low hospital visit rate during the pandemic. The proportion of visiting ophthalmologists in COVID-19-positive patients is almost half as the control group. It is possible that the prevalence of glaucoma is likely underestimated as individuals with COVID-19 have a range of manifestations and may be unlikely to seek out ophthalmic evaluation when other life-threatening conditions are present. However, in response to these challenges, several groups have implemented various techniques to remotely monitor and manage glaucoma patients during the pandemic (34–36). The diagnosis of cataract is more than twice as frequent in the COVID-19 negative group compared to the COVID-19 positive group. Although 25% of patients complain about ocular dryness, only 8% of those infected with COVID-19 were diagnosed as dry eye. The disparity between the diagnosed prevalence of symptoms and the low occurrence of dry eye in the COVID-19 positive group may be attributed to a lower rate of hospital visits.

There are several limitations in this study: as with any survey study, there was an unavoidable recall bias, and levels of symptom awareness varied among survey participants. There is also likely an inclusion bias toward respondents experiencing eye symptoms, since this survey was mainly distributed in the Northeast of China. As this online survey was anonymous, it is difficult to track the patients. Thus, we failed to collect the subtypes of glaucoma, uveitis, and ocular diseases. Another potential limitation is that our study may include factors other than SARS-CoV-2 infection, such as the emotional changes caused by the epidemic and the inconvenience of seeing a doctor. Lastly, a larger and more representative sample size would strengthen the findings of this study.

In conclusion, our results showed that ocular symptoms were related with systemic symptoms and the majority of participants reported the onset of ocular symptoms at the same time as systemic symptoms. Moreover, COVID-19 increased the prevalence of uveitis and optic neuritis, which should attract the attention of ophthalmologists and enhance the management of uveitis patients. We believe this study is critical to the understanding of ocular manifestations of COVID-19, particularly after the virus has undergone multiple mutations. Overall, further research is needed to fully understand the pathophysiology of ocular symptoms associated with COVID-19.

## Data Availability

The raw data supporting the conclusions of this article will be made available by the authors, without undue reservation.

## Supplementary material

SUPPLEMENTARY FIGURE 1 Correlation between ocular symptoms and vaccination status.

SUPPLEMENTARY FIGURE 2 Ocular manifestation in different vaccination status of participants.
