# Supplementary figures and images for: Characteristics of ocular findings of patients with coronavirus disease 2019 in China
Source: Front Med (Lausanne). 2025 Jan 13;11:1292821. doi: 10.3389/fmed.2024.1292821 (PMC11771207; doi:10.3389/fmed.2024.1292821)

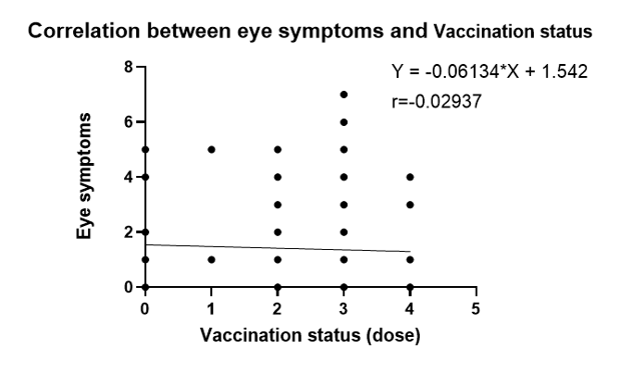

Supplement: SUPPLEMENTARY FIGURE 1 — Correlation between ocular symptoms and vaccination status. [file Image_1.tiff]

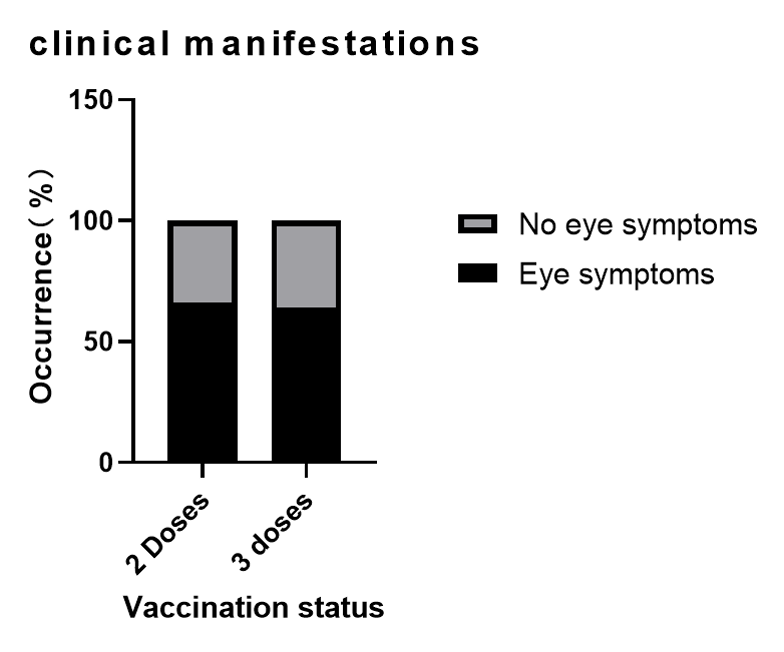

Supplement: SUPPLEMENTARY FIGURE 2 — Ocular manifestation in different vaccination status of participants. [file Image_2.tiff]
